# Short-Course Versus Prolonged-Course Antimicrobial Therapy in Adults With Catheter-Related Septic Thrombosis: A Propensity-Weighted Retrospective Study

**DOI:** 10.1093/ofid/ofad530

**Published:** 2023-10-25

**Authors:** Marine Stoldick, Margot Vannier, Mikael Verdalle-Cazes, Manuel Etienne, Kévin Alexandre

**Affiliations:** Department of Infectious Diseases, Centre hospitalier universitaire Rouen, Rouen, France; Department of Biostatistics, Centre hospitalier universitaire Rouen, Rouen, France; Department of Radiology, Centre hospitalier universitaire Rouen, Rouen, France; Univ Rouen Normandie, Université de Caen, INSERM, Normandie Univ, DYNAMICURE Unité mixte de recherche 1311, Centre hospitalier universitaire Rouen, Department of Infectious Diseases, Rouen, France; Univ Rouen Normandie, Université de Caen, INSERM, Normandie Univ, DYNAMICURE Unité mixte de recherche 1311, Centre hospitalier universitaire Rouen, Department of Infectious Diseases, Rouen, France

**Keywords:** catheter-related infection, duration, septic thrombosis, short-course therapy

## Abstract

**Background:**

Optimal duration of antimicrobial therapy (AT) for catheter-related septic deep venous thrombosis (DVT) is unknown. We aimed to compare the outcomes of patients receiving short-course AT (≤21 days) versus prolonged-course AT (>21 days).

**Methods:**

This was a monocentric retrospective study comparing adults with catheter-related septic DVT from 2015 to 2020 treated with short- or prolonged-course AT. A propensity score–weighted analysis was used to mitigate potential bias. The primary outcome was a composite of all-cause mortality or recurrent bloodstream infection 30 days after AT discontinuation.

**Results:**

Of 172 patients with catheter-related septic DVT, 104 were treated with prolonged-course AT and 68 with short-course AT. In the propensity score analysis, we found no significant difference in 30-day all-cause mortality or relapse between the 2 groups (inverse probability of treatment weighted hazard ratio [wHR], 2.16 [95% confidence interval {CI}, .68–6.88]; *P* = .192). No differences in 90-day all-cause mortality and 90-day relapse were observed between the treatment groups (wHR, 1.01 [95% CI, .49–2.05], *P* = .987 and 1.13 [95% CI, .08–15.62], *P* = .928, respectively).

**Conclusions:**

A 21-day AT could be an effective and safe option to treat catheter-related septic DVT. Further randomized studies are needed to establish the optimal duration of AT for patients with catheter-related septic DVT.

## Teaser

Optimal duration of antimicrobial therapy for catheter-related septic deep venous thrombosis (DVT) is unknown. According to this propensity score–weighted study on 172 patients, short-course antimicrobial therapy (≤21 days) could be an effective and safe option to treat catheter-related septic DVT.

Healthcare-associated primary bloodstream infections (BSIs) result in increased morbidity, mortality, and costs [1–3]. Indeed, every year in Europe the burden of healthcare-associated primary BSI is estimated at 25 000 deaths and 145 disability-adjusted life-years per 100 000 population [4]. Septic deep venous thrombosis (DVT) is a life-threatening infection complicating up to 70% of catheter-related bloodstream infections (CRBSIs) with an attributable mortality of approximately 20% [5, 6].

The recommended antimicrobial therapy (AT) duration of catheter-related septic DVT usually exceeds 4 weeks [7]. Exposure to such prolonged AT is associated with an increased likelihood of adverse events [8], *Clostridioides difficile* infections (CDIs) [9], and antibacterial resistance [10]. On the other hand, shorter AT durations allow a more rapid return to patient baseline activity [11] and reduce antibiotic exposure, venous access complications, and adverse events. Finding the optimal AT duration is challenging as shorter treatments may expose patients to relapse and death, particularly for pathogens such as *Staphylococcus aureus* and *Candida* spp [12–14].

Several trials have demonstrated no significant difference in mortality between short-course and prolonged-course AT in the treatment of gram-negative and gram-positive uncomplicated bacteremia [11, 15, 16].

The Infectious Diseases Society of America (IDSA) guidelines on septic DVT, based on expert opinion, recommend a prolonged AT of 3–6 weeks [7] for catheter-related septic DVT. In 2017, French experts proposed a unique treatment length of 3 weeks [17], relying on scarce data of such treatment reduction strategies.

In this context, we aimed to compare the effectiveness of short-course versus prolonged-course AT for catheter-related septic DVT of the upper limb.

## METHODS

### Study Setting and Design

This was a monocentric, retrospective study conducted between 2015 and 2020 in a 2500-bed teaching hospital in Rouen, France. Patients were eligible for inclusion if they were adults (aged ≥18 years) with a catheter-related septic DVT of the upper limb. Patients meeting any of the following criteria were excluded: missing information about AT duration, microbiological documentation or susceptibility testing, secondary septic localization (endocarditis, osteomyelitis, arthritis, and abscess), prolonged AT for another infection, and early death (ie, death within 21 days of the treatment initiation or on the day treatment ended). Patients for whom the catheter was not removed or with an infectious source different from the catheter (eg, bacteremic urinary infection with secondary septic thrombosis) were not excluded as long as the definition of catheter-related septic DVT was respected.

### Study Definitions

Catheter-related septic DVT was defined as the combination of a DVT homolateral to the catheter and certain or possible CRBSI.

DVT was defined as demonstration of a thrombus by sonography in a deep vein of the upper limb (subclavian, jugular, axillary, and humeral veins and the brachiocephalic venous trunk) with no size threshold.

Bacteremia was defined as ≥1 positive peripheral drawn blood culture.

Certain CRBSI was defined as bacteremia or fungemia in a patient with an intravascular device with 1 of the following criteria: (*i*) the same microbial species isolated from a quantitative catheter culture (ie, >10² colony-forming units [CFU]/mL) and ≥1 peripheral blood culture; (*ii*) growth of a microorganism in a blood culture obtained through a catheter hub detected by an automated blood culture system at least 2 hours earlier than a simultaneous peripheral drawn blood culture of equal volume [7].

Possible CRBSI was defined as the positive result of quantitative catheter culture (ie, >10^2^ CFU/mL) or growth in a culture of blood obtained through a catheter hub and systemic signs of infection (fever, chills, hypotension) with no other obvious source of infection [7, 18].

Long-term central venous catheters (CVCs) were defined as totally implanted access ports and tunneled catheters.

Short-term CVCs were defined as catheters inserted in a central vein used in critically ill patients or during anesthesia for a predicted duration of <14 days.

Other catheters were defined as midlines, peripherally inserted central catheters, hemodialysis catheters, or Swan-Ganz catheters.

### Data Collection

We screened patients using the hospital’s radiology software to identify every upper limb DVT over the study period. Then, we reviewed electronic and paper medical records to assess inclusion and exclusion criteria. The following data were collected: demographic data; underlying diseases (immunosuppression status [chemotherapy, immunotherapy, cirrhosis, transplantation, dialysis]); Charlson comorbidity-weighted index (CCWI); presence of permanent foreign device (ie, pacemaker, defibrillator, ventricular assist device, prosthetic heart valve, orthopedic device); microbiological results (possible or certain CRBSI, presence and duration of bacteremia, microorganisms); biological data (maximal C-reactive protein level and renal function); clinical presentation (fever, sepsis or septic shock, intensive care unit admission); thrombus characteristics (total or partial, number of venous segments involved [1 segment corresponding to 1 anatomic vein]); catheter characteristics (type, catheterization duration, indication); and management (catheter removal, oral treatment, curative anticoagulation). A public national registry recording all deaths of French citizens ascertained that all fatalities were taken into account in our study. We ensured that patients were followed up in our hospital, with no mention of relapse in either their medical or microbiological records. When patients were followed up in another hospital, we contacted them to make sure of the absence of relapse.

### Classification and Outcomes

Patients were classified as having received short-course or prolonged-course AT if they received ≤21 days or >21 days of AT, respectively. AT duration was defined as the duration of intravenous or oral AT between the first and the last day of appropriate AT (ie, proven in vitro susceptibility according to European Committee on Antimicrobial Susceptibility Testing breakpoints).

The primary outcome was a composite of 30-day posttreatment all-cause mortality or relapse, defined as positive blood culture(s) with the same species during the follow-up. Secondary outcomes were 90-day posttreatment all-cause mortality, relapse, CDI, a new infection or colonization with a more resistant or multidrug-resistant (MDR) bacteria, and the length of hospital stay. A more resistant bacteria was defined by the observation of a phenotypic resistance to an antibiotic to which the bacteria involved in the septic thrombosis was susceptible before exposure to AT. Systematic active surveillance for MDR bacteria was not performed in our institution.

### Patient Consent Statement

According to French law, no patient consent was required to collect and analyze retrospective and pseudonymized data. This study was approved by the Grading Committee of Research Projects from Rouen University Hospital (number 879).

### Statistical Analysis

Quantitative variables were described as mean and standard deviation or median and interquartile range (IQR). Categorical data were reported as numbers and percentages. The χ^2^ test or Fisher exact test was used for qualitative variables and Student *t* test or Mann-Whitney test for quantitative variables, as appropriate. A *P* value <.05 was considered statistically significant. Confidence intervals (CIs) were calculated at 95%.

Propensity scores (PSs) were calculated using a series of univariable Cox proportional hazard models to determine which patient characteristics were associated with the choice of treatment duration group. A covariate (patient characteristic) was included in the PS model if at least 1 of the following criteria was true for at least 1 of the outcomes: (*i*) the absolute value of the parameter estimate was at least 0.2 for at least 1 level of the covariate; or (*ii*) the *P* value was <.01 for at least 1 level of the covariate [19]. Applying these criteria, the following variables were included: age, sex, hospital ward, immunosuppression, CCWI, permanent foreign device, certain or possible CRBSI, presence of bacteremia, microorganism type, sepsis or septic shock, catheter type, time between the first microbiological documentation and catheter removal, time between the first microbiological documentation and initiation of effective AT, and curative anticoagulation. We assessed the balance of the covariates between short- and prolonged-course treatment groups using standardized differences and variance ratios. Standardized differences of <10% and variance ratios of <10% from 1.0 were considered negligible.

Weights were constructed as the inverse of the probability of being in the short-course group (1 / p) among those actually in the short-course group, and as the inverse of the probability of being in the prolonged-course group (1 / 1 – p) among those in the prolonged-course group.

We presented incidence of death or relapse 30 days and 90 days after treatment discontinuation on cumulative incidence curves with unweighting and inverse probability of treatment weighting (IPTW).

Cox proportional hazard models were used to compute hazard ratio (HR) and IPTW hazard ratio (wHR) with 95% CIs. The Hosmer-Lemeshow test was used to assess the fit of regression models.

Sensitivity analysis was performed by including only patients with a certain CRBSI. The following variables were included in the PS: age, sex, hospital ward, immunosuppression, CCWI, permanent foreign device, microorganism type, sepsis or septic shock, catheter type, time between the first microbiological documentation and catheter removal, time between the first microbiological documentation and initiation of effective AT, and curative anticoagulation.

Data analysis was performed using SAS Enterprise Guide version 8.1 software.

## RESULTS

### Patient Characteristics

**Figure 1.**
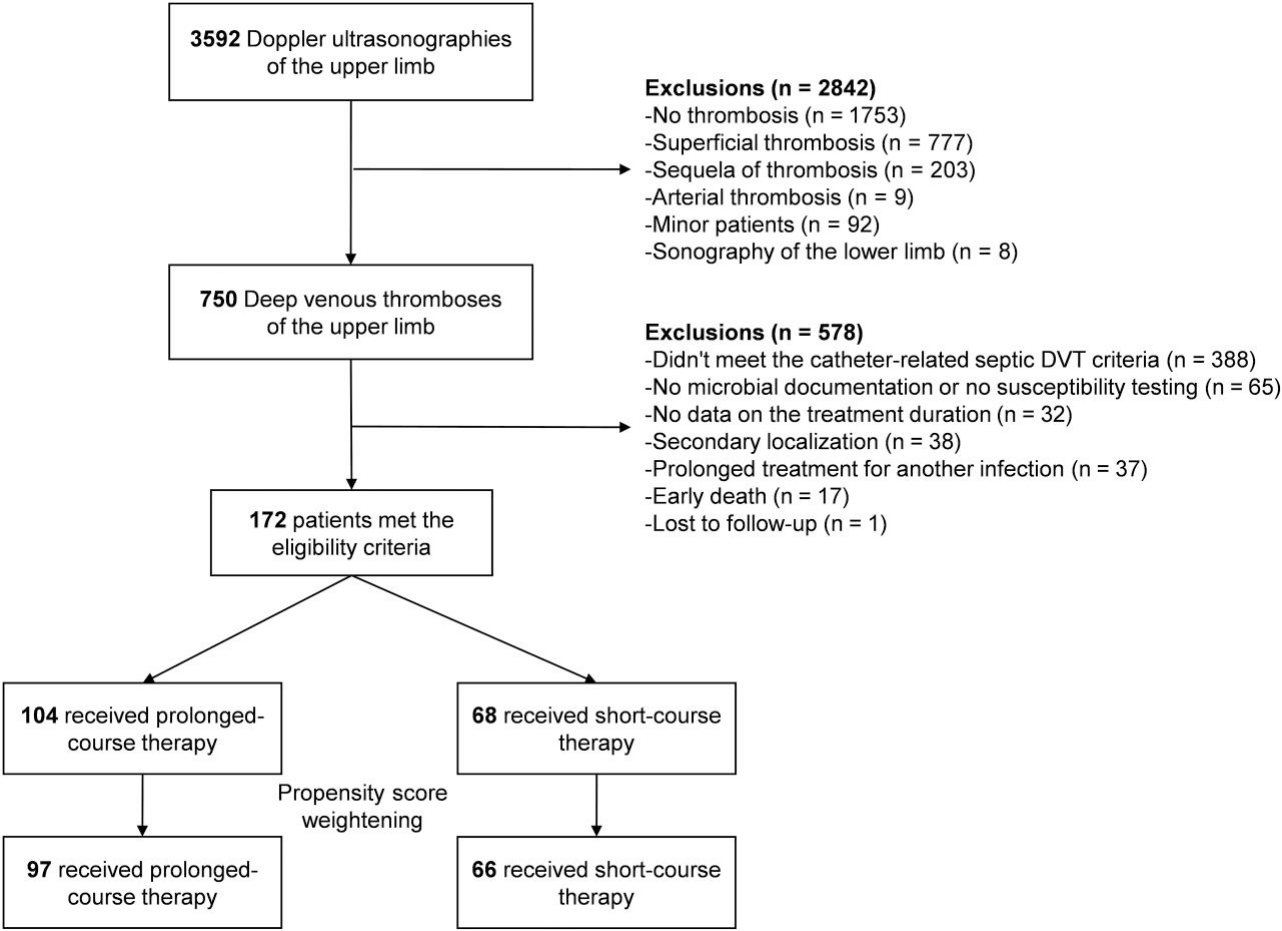
Study flowchart. Abbreviation: DVT, deep venous thrombosis.

Between January 2015 and December 2020, 3592 Doppler ultrasonography reports were reviewed, finding 750 DVTs of the upper limb. After exclusion, data from 172 adult patients were analyzed (68 in the short-course AT group and 104 in the prolonged-course AT group). Finally, 163 patients were analyzed with the PS, as data were missing for 4 patients regarding the covariate “curative anticoagulation” and for 5 patients regarding the covariate “time between the first microbiological documentation and catheter removal” (Figure 1).

Patient characteristics are presented in Table 1. The median age was 63 (IQR, 52.5–70) years, 73 patients (42.4%) were female, and the median overall CCWI was 5 (IQR, 3–8). The median duration of AT was 16 (IQR, 13–21) days in the short-course group and 30 (IQR, 25.5–42) days in the prolonged-course group. As expected, the majority of short-course treatments were administered after 2017. Baseline characteristics displayed some significant differences: sex, hospital ward, immunosuppression, presence of permanent foreign device, possible or certain CRBSI, presence of bacteremia, type of catheter, time between the first microbiological documentation and catheter removal, time between the first microbiological documentation and initiation of effective AT, and curative anticoagulation. Duration of curative anticoagulation was unavailable for 45 patients. Among the 98 other patients, 39% received ≤6 weeks of curative anticoagulation, 37% received >6 weeks, and 24% were under long-term anticoagulation.

**Table 1.** Baseline Characteristics of Adults With Catheter-Related Septic Deep Venous Thrombosis Receiving Short- or Prolonged-Course Antimicrobial Therapy

Characteristic	Total (N = 172)	Short-Course (n = 68)	Prolonged-Course (n = 104)	*P* Value
Patient general status				
Age, y, median (IQR)	63 (52.5–70)	65 (53–70.5)	63 (51.5–69)	.499
Female sex	73 (42.4)	22 (32.3)	51 (49.0)	.030
BMI (n = 169), kg/m^2^, mean (SD)	24.8 (6.2)	25.1 (5.4)	24.6 (6.7)	.586
Surgical ward	67 (39.2)	36 (52.9)	31 (30.1)	.003
Medical ward	104 (60.8)	32 (47.1)	72 (69.9)	
Period				
2014–2017	66 (38.4)	20 (29.4)	46 (44.2)	.050
2018–2020	106 (61.6)	48 (70.6)	58 (55.8)	
Underlying conditions				
Solid tumor	93 (54.1)	40 (58.8)	53 (51.0)	.312
Hematologic malignancy	6 (3.5)	3 (4.4)	3 (2.9)	.682
Immunocompromised	67 (39.0)	20 (29.4)	47 (45.2)	.038
Palliative care	11 (6.4)	3 (4.4)	8 (7.7)	.530
CCWI, median (IQR)	5 (3–8)	6 (3–8)	5 (2–8)	.592
Permanent foreign device	20 (11.6)	13 (19.1)	7 (6.7)	.013
Microbiological data				
Certain CRBSI	116 (67.4)	35 (51.5)	81 (77.9)	.0003
Bacteremia	128 (74.4)	40 (58.8)	88 (84.6)	.0003
Bacteremia duration (n = 120), d, median (IQR)	4 (2–6)	4 (2–5)	4 (2–6)	.377
CoNS	61 (35.5)	27 (39.7)	34 (32.7)	
*Staphylococcus aureus*	39 (22.7)	9 (13.2)	30 (28.9)	
GNB	17 (9.9)	6 (8.8)	11 (10.6)	
*Candida* spp	13 (7.6)	5 (7.4)	8 (7.7)	
NFGNB	9 (5.2)	2 (2.9)	7 (6.7)	
Others	33 (19.2)	19 (27.9)	14 (13.5)	
Biological data				
Creatinine, µmol/L, median (IQR)	67 (49–95.5)	67 (46–89)	70 (50–99)	.180
Maximal CRP (n = 166), mg/L, median (IQR)	109.5 (55–192)	93.5 (51.5–190.5)	113 (57–192)	.701
Clinical data				
Fever (n = 152)	147 (96.7)	62 (98.4)	85 (95.5)	.404
Sepsis or septic shock	28 (16.3)	8 (11.8)	20 (19.2)	.195
ICU admission	26 (15.1)	10 (14.7)	16 (15.4)	.903
Thrombosis				
Total thrombosis (n = 153)	14 (10.0)	5 (8.5)	9 (11.1)	.608
>1 venous segment	24 (14.0)	8 (11.8)	16 (15.4)	.503
Catheter				
Type				
Short-term CVC	63 (36.6)	36 (52.9)	27 (26.0)	.001
Long-term CVC	53 (30.8)	13 (19.1)	40 (38.4)	
Other catheters	56 (32.6)	19 (28.0)	37 (35.6)	
Duration (n = 145), d, median (IQR)	15 (8–28)	15.5 (8–26)	14 (7–31)	.968
Indication				
Parenteral nutrition	34 (20.7)	14 (21.5)	20 (20.2)	.248
Chemotherapy	47 (28.7)	14 (21.5)	33 (33.3)	
Other	83 (50.6)	37 (57.0)	46 (46.5)	
Management				
Catheter removal	167 (97.1)	67 (98.5)	100 (96.2)	
AT duration, d, median (IQR)	24.5 (19–31)	16 (13–21)	30 (25.5–42)	<.0001
AT duration >14 d	147 (85.5)	43 (63.2)	104 (100)	
Exclusive oral treatment	4 (2.3)	3 (4.4)	1 (1.0)	.302
Oral switch	17 (9.9)	7 (10.3)	10 (9.6)	.884
Curative anticoagulation (n = 168)	143 (85.1)	52 (77.6)	91 (90.1)	.026
Anticoagulation duration (n = 143)				.063
≤6 wk	38 (26.6)	8 (15.4)	30 (33.0)	
>6 wk	36 (25.2)	14 (26.9)	22 (24.2)	
Long-term anticoagulation	24 (16.8)	13 (25)	11 (12.1)	
Unknown	45 (31.5)	17 (32.7)	28 (30.8)	

Data are presented as No. (%) unless otherwise indicated.

Abbreviations: AT, antimicrobial therapy; BMI, body mass index; CCWI, Charlson comorbidity-weighted index; CoNS, coagulase-negative staphylococci; CRBSI, catheter-related bloodstream infection; CRP, C-reactive protein; CVC, central venous catheter; GNB, gram-negative bacilli; ICU, intensive care unit; IQR, interquartile range; NFGNB, nonfermenting gram-negative bacilli; SD, standard deviation.

The proportion of *S aureus*, *Candida* spp, and nonfermenting gram-negative bacilli (NFGNB) was higher in the prolonged-course group (43.3% vs 23.5%), with a clear imbalance concerning *S aureus* infection (28.9% vs 13.2%). Overall, the most frequently isolated species were coagulase-negative staphylococci (35.5%), followed by *S aureus* (22.7%), gram-negative bacilli (9.9%), *Candida* spp (7.6%), and NFGNB (5.2%). Among the 56 possible CRBSIs, 33 (59%) presented clinical symptoms (purulence or inflammation) or the diagnosis was confirmed by an infectious diseases specialist.

### Primary Outcome

**Figure 2.**
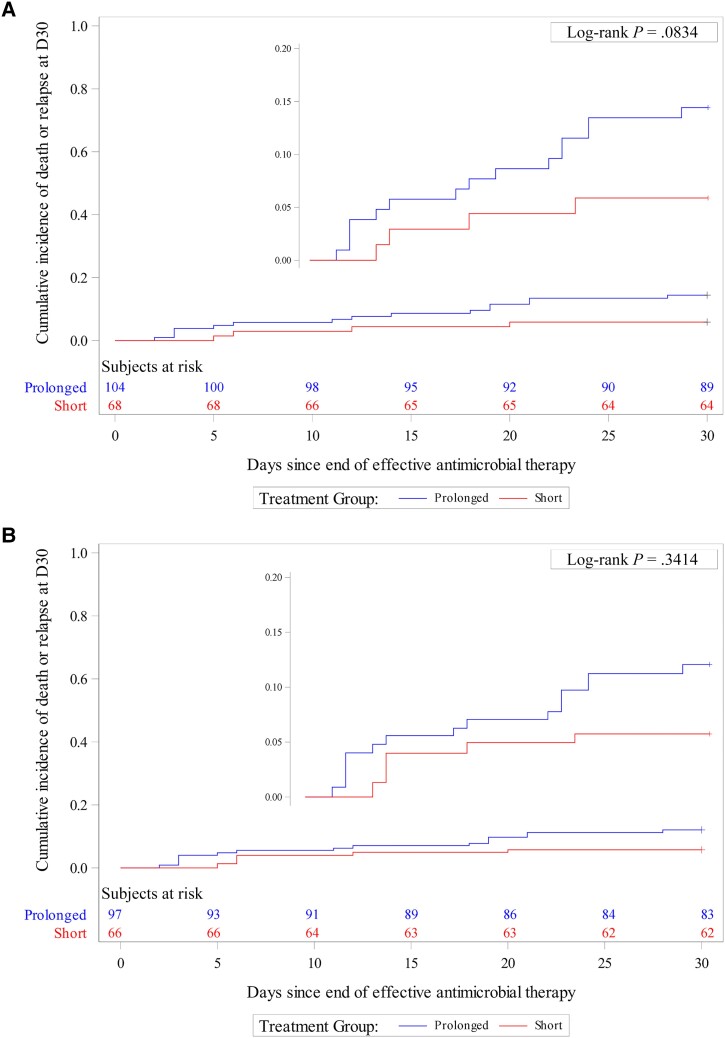
Cumulative event curves for 30-day all-cause mortality or relapse in the unweighted (*A*) and inverse probability of treatment weighted (*B*) samples in the total population.

In both unweighted and weighted analyses, we found no significant difference in terms of 30-day all-cause mortality or relapse between short and prolonged AT durations. In the unweighted analyses, by day 30 posttreatment, 4 of 68 (5.9%) and 15 of 104 (14.4%) patients died or experienced relapse in the short-course and prolonged-course groups, respectively (HR, 2.55 [95% CI, .85–7.69]; *P* = .096) (Table 2, Figure 2*A*). After PS weighting, on day 30 posttreatment, 4 of 66 (6.1%) and 14 of 97 (14.4%) patients died or experienced relapse in the short-course and prolonged-course groups, respectively (wHR, 2.16 [95% CI, .68–6.88]; *P* = .192) (Table 2, Figure 2*B*). The c-statistic value of the PS model was 0.807.

**Table 2.** Hazard Ratios of 30-Day All-Cause Mortality or Relapse, 90-Day All-Cause Mortality, and 90-Day Relapse in Patients Treated With Short- and Prolonged-Course Antimicrobial Therapy, Before and After Propensity Score Weighting According to Catheter-Related Bloodstream Infection Diagnosis

All-Cause Mortality or Relapse	Total Population (Possible and Certain CRBSIs)				Sensitivity Analysis (Certain CRBSIs)			
	HR (95% CI) (n = 172)	*P* Value	wHR (95% CI) (n = 163)	*P* Value	HR (95% CI) (n = 116)	*P* Value	wHR (95% CI) (n = 113)	*P* Value
30-d all-cause mortality or relapse	2.55 (.85–7.69)	.096	2.16 (.68–6.88)	.192	1.92 (.55–6.72)	.310	2.10 (.54–8.20)	.286
90-d all-cause mortality	1.52 (.74–3.09)	.253	1.01 (.49–2.05)	.987	1.28 (.50–3.24)	.604	1.12 (.43–2.89)	.819
90-d relapse	0.68 (.10–4.86)	.705	1.13 (.08–15.62)	.928	0.45 (.06–3.18)	.423	1.14 (.07–18.81)	.928

Abbreviations: AT, antimicrobial therapy; CI, confidence interval; CRBSI, catheter-related bloodstream infection; HR, hazard ratio; wHR, inverse probability of treatment weighted hazard ratio.

In univariate analysis, short-course AT duration was not associated with 30-day all-cause mortality or relapse (*P* = .081) (Supplementary Table 1). Also, infections due to *S aureus*, *Candida* spp, or NFGNB were not associated with a difference in the primary outcome (*P* = .619) (Supplementary Table 1). Palliative care, hospitalization in a medical ward, catheter type, indication for catheter placement, and time between the first microbiological documentation and catheter removal were significantly associated with an increased risk of 30-day mortality or relapse (Table 3). In multivariate analysis, these associations were not significant (Table 3).

**Table 3.** Significant Risk Factors of 30-Day All-Cause Mortality or Relapse in Univariate and Multivariate Analysis According to Catheter-Related Bloodstream Infection Diagnosis

Risk Factor	Total Population (Possible and Certain CRBSIs) (n = 172)				Sensitivity Analysis (Certain CRBSIs) (n = 116)			
	Univariate Analysis		Multivariate Analysis		Univariate Analysis		Multivariate Analysis	
	OR (95% CI)	*P* Value	OR (95% CI)	*P* Value	OR (95% CI)	*P* Value	OR (95% CI)	*P* Value
Hospital ward	n = 171		n = 171		n = 116		n = 116	
Surgical	Reference		Reference		Reference		Reference	
Medical	6.35 (1.42–28.46)	**.016**	2.20 (.39–12.40)	.373	4.67 (1.01–21.65)	**.049**	1.58 (.23–10.79)	.638
Palliative care	n = 172		n = 172		n = 116		n = 116	
No	Reference		Reference		Reference		Reference	
Yes	8.75 (2.37–32.35)	**.001**	4.51 (.95–21.34)	.057	16.33 (2.70–98.82)	**.002**	6.49 (.63–66.29)	.115
Catheter type	n = 172		n = 172		n = 116		n = 116	
Short-term CVC	Reference		Reference		Reference		Reference	
Long-term CVC	12.68 (1.55–103.75)	**.018**	3.20 (.21–48.59)	.401	10.85 (1.28–91.78)	**.029**	1.36 (.06–31.43)	.849
Other catheters	11.87 (1.45–96.99)	**.021**	5.06 (.41–61.85)	.204	7.82 (.91–66.83)	.060	1.88 (.11–32.97)	.666
Catheter indication	n = 164		n = 164		n = 111		n = 111	
Chemotherapy	Reference		Reference		Reference		Reference	
Parenteral nutrition	0.56 (.16–2.00)	.376	0.61 (.13–2.77)	.519	0.36 (.09–1.53)	.169	0.40 (.07–2.22)	.298
Other	0.21 (.06–.74)	**.015**	0.61 (.13–2.83)	.532	0.10 (.02–.48)	**.004**	0.20 (.02–1.98)	.170
Time between the first microbiological documentation and catheter removal	n = 167		n = 167		n = 114		n = 114	
	1.12 (1.01–1.25)	**.032**	1.06 (.91–1.22)	.462	1.14 (1.01–1.30)	**.041**	1.05 (.86–1.29)	.609

*P* value in bold: *P* < .05. Abbreviations: CI, confidence interval; CRBSI, catheter-related bloodstream infection; CVC, central venous catheter; OR, odds ratio.

### Secondary Outcomes

Using the PS, we found no significant difference between short-course and prolonged-course AT groups for 90-day all-cause mortality (11/66 [16.7%] vs 21/97 [21.6%], respectively; wHR, 1.01 [95% CI, .49–2.05]; *P* = .987) and 90-day relapse (1/66 [1.5%] vs 2/97 [2.1%], respectively; wHR, 1.13 [95% CI, .08–15.62]; *P* = .928) (Table 2, Supplementary Figures 1*B* and 3*B*). There were 2 (2.9%) CDIs in the short-course AT group whereas none occurred in the prolonged-course AT group (not significant, *P* = .155; Table 4). There were 2 (2.9%) reports of new colonization or infection with a more resistant or MDR organism in the short-course group and 5 (4.8%) in the prolonged-course group (not significant, *P* = .705; Table 4). The length of hospital stay was not significantly different between the 2 groups, with a median of 33 days and 30 days in the short-course and the prolonged-course groups, respectively (*P* = .536; Table 4).

**Table 4.** Proportion of 90-Day *Clostridioides difficile* Infection and 90-Day Colonization or Infection with a More Resistant or Multidrug-Resistant Organism and Length of Hospital Stay in Patients Treated With Short- and Prolonged-Course Antimicrobial Therapy According to Catheter-Related Bloodstream Infection Diagnosis

Secondary Outcomes	Total Population (Possible and Certain CRBSIs) (n = 172)			Sensitivity Analysis (Certain CRBSIs) (n = 116)		
	Short-Course (n = 68)	Prolonged-Course (n = 104)	*P* Value	Short-Course (n = 68)	Prolonged-Course (n = 104)	*P* Value
90-d CDI	2 (2.9%)	0 (0.0%)	.155	1 (2.9%)	0 (0.0%)	.302
90-d colonization or infection with a more resistant or MDR organism	2 (2.9%)	5 (4.8%)	.705	1 (2.9%)	5 (6.2%)	.666
Length of hospital stay (n = 170), d, median (IQR)	33.0 (17–58.0)	30.0 (19.0–48.0)	.536	36.0 (17–79.0)	30.0 (19.0–49.0)	.359

Abbreviations: AT, antimicrobial therapy; CDI, *Clostridioides difficile* infection; CRBSI, catheter-related bloodstream infection; IQR, interquartile range; MDR, multidrug resistant.

### Sensitivity Analysis

Data from 116 adult patients with certain CRBSI were analyzed: 35 in the short-course AT group and 81 in the prolonged-course AT group. Finally, 113 patients were analyzed with the PS as data were missing for 1 patient regarding the covariate “curative anticoagulation” and for 2 patients regarding the covariate “time between the first microbiological documentation and catheter removal.” Patient characteristics are presented in Supplementary Table 2.

**Figure 3.**
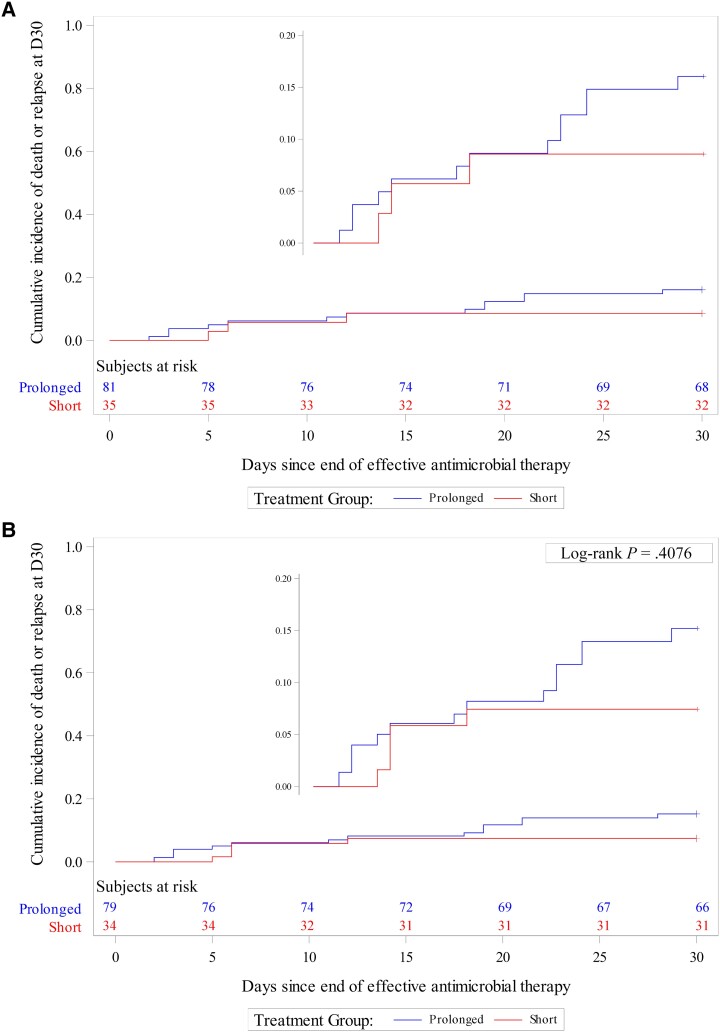
Cumulative event curves for 30-day all-cause mortality or relapse in the unweighted (*A*) and inverse probability of treatment weighted (*B*) samples in patients with certain catheter-related bloodstream infections.

Performing our analyses including only patients with certain CRBSI had no effect on the findings of the study. In both unweighted and weighted analyses, we found no significant difference in terms of 30-day all-cause mortality or relapse between short and prolonged AT durations. In the unweighted analyses, by day 30 posttreatment, 3 of 35 (8.6%) and 13 of 81 (16.0%) patients died or experienced relapse in the short-course and prolonged-course groups, respectively (HR, 1.92 [95% CI, .55–6.72]; *P* = .310) (Table 2, Figure 3*A*). After propensity weighting, on day 30 posttreatment, 3 of 34 (8.8%) and 13 of 79 (16.5%) patients died or experienced relapse in the short-course and prolonged-course groups, respectively (wHR, 2.10 [95% CI, .54–8.20]; *P* = .286) (Table 2, Figure 3*B*). The c-statistic value of the PS model was 0.778.

In univariate analysis, short-course AT duration was not associated with 30-day all-cause mortality or relapse (*P* = .385) (Supplementary Table 3). Also, infections due to *S aureus*, *Candida* spp, or NFGNB were not associated with a difference in the primary outcome (*P* = .663) (Supplementary Table 3). Palliative care, hospitalization in a medical ward, catheter type, indication for catheter placement, and time between the first microbiological documentation and catheter removal were significantly associated with an increased risk of 30-day mortality or relapse (Table 3). In multivariate analysis, these associations were not significant (Table 3).

Using the PS, we found no significant difference between short and prolonged AT groups for 90-day all-cause mortality (6/34 [17.6%] vs 16/79 [20.3%], respectively; wHR, 1.12 [95% CI, .43–2.89]; *P* = .819) and 90-day relapse (1/34 [2.9%] vs 2/79 [2.5%], respectively; wHR, 1.14 [95% CI, .07–18.81]; *P* = .928) (Table 2, Supplementary Figures 2*B* and 4*B*). There was 1 (2.9%) CDIs in the short-course AT group, whereas none occurred in the prolonged-course AT group (not significant, *P* = .302; Table 4). There was 1 (2.9%) reports of new colonization or infection with a more resistant or MDR organism in the short-course group and 5 (6.2%) in the prolonged-course group (not significant, *P* = .666; Table 4). The length of hospital stay was not significantly different between the 2 groups, with a median of 36 days and 30 days in the short-course and the prolonged-course groups, respectively (*P* = .359; Table 4).

## DISCUSSION

In this monocentric propensity-weighted retrospective study, we found that patients receiving ≤21 days of AT for catheter-related septic DVT have a similar risk of death and relapse within 30 days after treatment discontinuation as patients receiving prolonged-course AT.

Current IDSA guidelines for catheter-related septic DVT suggest prescribing AT for a total length of 3–6 weeks, based on a low quality of evidence due to scarcity of studies on this subject [7]. Some experts suggest a unique treatment duration as long as 3 weeks [17]. The microbiological epidemiology in our study was in accordance with previous studies [6, 20], and the PS allowed us to reduce the effects of some assessed confounding factors and to balance out the differences between the 2 groups, in particular the higher proportion of *S aureus* and NFGNB infection in the prolonged-course group. Nevertheless, we were not able to analyze the outcomes according to microorganisms due to the size of study. Furthermore, some data suggest that treatment duration reduction should not be done in specific populations or for particular microbial species. Indeed, shorter-course AT (14 days) for *S aureus* uncomplicated CRBSI is not recommended for immunosuppressed patients due to an increased risk of death [7]. Wilson Dib et al found that overall mortality rate was significantly lower for patients receiving ≥28 days of AT for catheter-related *S aureus* DVT than patients receiving <28 days of AT [13]. In *Candida* septic thrombosis, AT duration in survivors is always prolonged (>4 weeks), whereas fatal cases have received shorter-course AT as described by Strinden et al in 8 reported cases [14]. In our study, only 13 patients suffered from catheter-related septic DVT due to *Candida* spp. Therefore, shortening AT duration for fungal and *S aureus* infections should be done with caution considering these limited data. Additionally, oncohematologic patients were underrepresented because of our monocentric design, excluding hematologic patients treated in a different hospital.

Possible CRBSIs were included despite the risk of lower specificity of this definition, as for half of these infections, we found additional arguments to enhance diagnostic performance (clinical symptoms or confirmed diagnosis by an infectious diseases specialist). Fewer patients with certain CRBSI-DVT diagnoses were noticed in the short-course AT group and therefore possibly more nonseptic thromboses were treated in this group, leading to the lack of difference observed in the study. However, we have taken these limitations into account by performing a sensitivity analysis based only on certain CRBSIs. Nevertheless, confirming the septic nature of a thrombus is challenging as its microbiological culture is almost never realized. Some authors suggest using positron emission tomography–computed tomography [PET/CT]) in order to confirm septic thrombosis in cases of bacteremia associated with catheter-related DVT. In a retrospective study, Gompelman et al found that 90-day all-cause mortality or relapse was not significantly different in patients with nonremarkable [^18^F]FDG (2-[^18^F]fluoro-2-deoxy-D-glucose)–PET/CT uptake receiving 14-day AT or oral switch therapy, compared to patients treated with prolonged-course intravenous AT for *S aureus* catheter-related DVT [21]. Nevertheless, due to prevalence of catheter-related DVT, cost and accessibility of PET such as [^18^F]FDG-PET/CT-guided AT duration should be prospectively evaluated.

Reduction of AT duration is a keystone of antimicrobial stewardship, considering the potential risks of excessive AT duration: CDI, new resistance, and longer hospital stays [9, 22, 23]. Due to low incidence of CDI and MDR bacteria, our study did not provide sufficient evidence of improvement of these metrics with shorter-course compared to prolonged-course AT. The similar length of hospital stay in both groups could have been explained by a more detailed analysis of the reasons for hospitalization to assess the proportion of hospital stay attributable to septic thrombosis and its treatment and that attributable to other conditions in this oncologic population. Moreover, outpatient parenteral AT could have reduced the length of hospital stay, despite a longer duration of antibiotic therapy.

The major limitation of our study is its retrospective design. Potential selection biases may have occurred in that some prognostic factors may have influenced treatment decision, such as clinical severity, which we could not accurately characterize with scoring. We tried to overcome these biases with the use of a PS-weighted analysis, and groups were well-balanced in IPTW analysis. However, we cannot exclude the possibility of additional, unmeasured confounding factors that could impact the association between AT duration and mortality or relapse. Thanks to available death records from the national registry, we had no missing data concerning all-cause mortality and we made sure that there was a regular hospital follow-up to monitor the absence of relapse.

Future fields of study could focus on the development of better diagnostic methods of septic thrombosis using nuclear imaging methods. Moreover, wide variability in AT duration suggests a need to produce high-quality evidence to identify optimal AT duration according to etiology microorganism. Further prospective and randomized trials are needed to validate our findings.

In conclusion, our study provides interesting data regarding optimal treatment duration of catheter-related septic DVT. A 21-day AT for such infections appears as safe as longer-course AT. Nevertheless, pending additional studies, consideration should be given to prolonged treatment durations in patients with *Candida* spp septic DVT and in oncohematologic patients.

## Supplementary Material

ofad530_Supplementary_Data

## Supplementary Data

Supplementary materials are available at *Open Forum Infectious Diseases* online. Consisting of data provided by the authors to benefit the reader, the posted materials are not copyedited and are the sole responsibility of the authors, so questions or comments should be addressed to the corresponding author.

## Notes

***Acknowledgments.*** The authors are grateful to Anaïs Lesourd for her support in editing the manuscript.
