# Supplementary material for: Short-Course Versus Prolonged-Course Antimicrobial Therapy in Adults With Catheter-Related Septic Thrombosis: A Propensity-Weighted Retrospective Study
Source: Open Forum Infect Dis. 2023 Oct 25;10(11):ofad530. doi: 10.1093/ofid/ofad530 (PMC10638491; doi:10.1093/ofid/ofad530)
Supplement: ofad530_Supplementary_Data [file ofad530_supplementary_data.docx]

SUPPLEMENTARY DATA

Supplementary Table S1. Risk factors of 30-day all-cause mortality or relapse in univariate analysis in total population.

|  | **Total**  **n=172** | **No death or relapse at D-30**  **n=153** | **Death or relapse at D-30**  **n=19** | **p value** |
| --- | --- | --- | --- | --- |
| **Treatment group** |  |  |  |  |
| Short-course | 68 (39.5) | 64 (41.8) | 4 (21.1) | .081 |
| Prolonged-course | 104 (60.5) | 89 (58.2) | 15 (78.9) |  |
| **Patient general status** |  |  |  |  |
| Age, years, median (IQR) | 63 (52.5-70) | 63 (53-69) | 67 (51-74) | .424 |
| Gender |  |  |  |  |
| Female | 73 (42.4) | 65 (42.5) | 8 (42.1) |  |
| Male | 99 (57.6) | 88 (57.5) | 11 (57.9) | .975 |
| BMI (n=169), kg/m², mean (SD) | 24.8 (6.2) | 25.0 (6.3) | 22.7 (5.3) | .100 |
| Hospital ward (n=171) |  |  |  |  |
| Surgical ward | 67 (39.2) | 65 (42.8) | 2 (10.5) | **.007** |
| Medical ward | 104 (60.8) | 87 (57.2) | 17 (89.5) |  |
| Period |  |  |  |  |
| 2014-2017 | 66 (38.4) | 60 (39.2) | 6 (31.6) | .519 |
| 2018-2020 | 106 (61.6) | 93 (60.8) | 13 (68.4) |  |
| **Underlying conditions** |  |  |  |  |
| Solid tumor |  |  |  |  |
| Yes | 93 (54.1) | 80 (52.3) | 13 (68.4) | .183 |
| No | 79 (45.9) | 73 (47.7) | 6 (31.6) |  |
| Hematologic malignancy |  |  |  |  |
| Yes | 6 (3.5) | 6 (3.9) | 0 (0) | / |
| No | 166 (96.5) | 147 (96.1) | 19 (100) |  |
| Immunocompromised |  |  |  |  |
| Yes | 67 (39.0) | 57 (37.3) | 10 (52.6) | .195 |
| No | 105 (61.0) | 96 (62.7) | 9 (47.4) |  |
| Palliative care |  |  |  |  |
| Yes | 11 (6.4) | 6 (3.9) | 5 (26.3) | **.003** |
| No | 161 (93.6) | 147 (96.1) | 14 (73.7) |  |
| CCWI, median (IQR) | 5 (3-8) | 5 (3-8) | 7 (5-8) | .104 |
| Permanent foreign device |  |  |  |  |
| Yes | 20 (11.6) | 19 (12.4) | 1 (5.3) | .702 |
| No | 152 (88.4) | 134 (87.6) | 18 (94.7) |  |
| **Supplementary Table S1. *(continued)***  **Microbiological data** |  |  |  | ***(continued)*** |
| CRBSI |  |  |  |  |
| Certain | 116 (67.4) | 100 (65.4) | 16 (84.2) | .098 |
| Possible | 56 (32.6) | 53 (34.6) | 3 (15.8) |  |
| Bacteremia |  |  |  |  |
| Yes | 128 (74.4) | 112 (73.2) | 16 (84.2) | .408 |
| No | 44 (25.6) | 41 (26.8) | 3 (15.8) |  |
| Bacteremia duration (n=120), days, median (IQR) | 4 (2-6) | 4 (2-6) | 4 (3.5-5) | .625 |
| Microorganism |  |  |  |  |
| *S. aureus* | 39 (22.7) | 36 (23.5) | 3 (15.8) | .619 |
| *Candida* spp. | 13 (7.6) | 12 (7.8) | 1 (5.3) |  |
| NFGNB | 9 (5.2) | 7 (4.6) | 2 (10.5) |  |
| Others | 111 (64.5) | 98 (64.1) | 13 (68.4) |  |
| **Biological data** |  |  |  |  |
| Creatinin, µmol/L, median (IQR) | 67 (49-95.5) | 67 (49-95) | 80 (52-106) | .331 |
| Maximal CRP (n=166), mg/L, median (IQR) | 109.5 (55-192) | 110.5 (54.5-193.5) | 92.5 (63-187) | .977 |
| **Clinical data** |  |  |  |  |
| Fever (n=152) |  |  |  |  |
| Yes | 147 (96.7) | 128 (96.2) | 19 (100) | / |
| No | 5 (3.3) | 5 (3.8) | 0 (0) |  |
| Sepsis or septic shock |  |  |  |  |
| Yes | 28 (16.3) | 23 (15.0) | 5 (26.3) | .202 |
| No | 144 (83.7) | 130 (85.0) | 14 (73.7) |  |
| ICU admission |  |  |  |  |
| Yes | 26 (15.1) | 23 (15.0) | 3 (15.8) | 1 |
| No | 146 (84.9) | 130 (85.0) | 16 (84.2) |  |
| **Thrombosis** |  |  |  |  |
| Total thrombosis (n=140) |  |  |  |  |
| Yes | 14 (10.0) | 14 (11.2) | 0 (0) | / |
| No | 126 (90.0) | 111 (88.8) | 15 (100) |  |
| >1 venous segment |  |  |  |  |
| Yes | 24 (14.0) | 20 (13.1) | 4 (21.1) | .310 |
| No | 148 (86.0) | 133 (86.9) | 15 (78.9) |  |
| **Catheter** |  |  |  |  |
| Type |  |  |  |  |
| Other catheters | 56 (32.6) | 47 (30.7) | 9 (47.4) | **.011** |
| Short-term CVC | 63 (36.6) | 62 (40.5) | 1 (5.3) |  |
| Long-term CVC | 53 (30.8) | 44 (28.8) | 9 (47.4) |  |
| Duration (n=145), days, median (IQR)  **Supplementary Table S1. *(continued)*** | 15 (8-28) | 14 (8-28) | 23 (8-59) | .450  ***(continued)*** |
| Indication |  |  |  |  |
| Parenteral nutrition | 34 (20.7) | 30 (20.4) | 4 (23.5) | **.035** |
| Chemotherapy | 47 (28.7) | 38 (25.9) | 9 (52.9) |  |
| Other | 83 (50.6) | 79 (53.7) | 4 (23.5) |  |
| **Management** |  |  |  |  |
| Catheter removal |  |  |  |  |
| Yes | 167 (97.1) | 148 (96.7) | 19 (100) | / |
| No | 5 (2.9) | 5 (3.3) | 0 (0) |  |
| Time between the first microbiological documentation and catheter removal (n=167) | 1 (0-3) | 1 (0-3) | 3 (1-7) | **.018** |
| Time between the first microbiological documentation and initiation of effective AT | 1 (0-3) | 1 (0-3) | 1 (0-2) | .705 |
| AT duration, days, median (IQR) | 24.5 (19-31) | 24 (17-31) | 28 (23-37) | .129 |
| AT duration > 14 days |  |  |  |  |
| Yes | 147 (85.5) | 128 (83.7) | 19 (100) | / |
| No | 25 (14.5) | 25 (16.3) | 0 (0) |  |
| Exclusive oral treatment |  |  |  |  |
| Yes | 4 (2.3) | 4 (2.6) | 0 (0) | / |
| No | 168 (97.7) | 149 (97.4) | 19 (100) |  |
| Oral switch |  |  |  |  |
| Yes | 17 (9.9) | 15 (9.8) | 2 (10.5) | 1 |
| No | 155 (90.1) | 138 (90.2) | 17 (89.5) |  |
| Curative anticoagulation (n=168) |  |  |  |  |
| Yes | 143 (85.1) | 127 (84.7) | 16 (88.9) | 1 |
| No | 25 (14.9) | 23 (15.3) | 2 (11.1) |  |
| Anticoagulation duration (n=143) |  |  |  |  |
| ≤ 6 weeks | 38 (26.6) | 35 (27.6) | 3 (18.8) | .086 |
| > 6 weeks | 36 (25.1) | 35 (27.6) | 1 (6.2) |  |
| Long-term anticoagulation | 24 (16.8) | 21 (16.5) | 3 (18.8) |  |
| Unknown | 45 (31.5) | 36 (28.3) | 9 (56.2) |  |

Data are presented as numbers (%) unless otherwise indicated.

Abbreviations: IQR: Interquartile range; SD: standard deviation; BMI: body mass index; CCWI: Charlson comorbidity weighted-index; CRP: C-reactive protein; CRBSI: catheter-related bloodstream infection; CoNS: coagulase-negative staphylococci; GNB: Gram-negative bacilli; NFGNB: non-fermenting Gram-negative bacilli; DVT: deep venous thrombosis; ICU: intensive care unit; CVC: central venous catheter; AT: antimicrobial therapy.

Supplementary Table S2. Baseline characteristics of adults with certain catheter-related septic deep venous thrombosis receiving short- or prolonged-course of AT.

|  | **Total**  **n=116** | **Short-course**  **n=35** | **Prolonged-course**  **n=81** | **p value** |
| --- | --- | --- | --- | --- |
| **Patient general status** |  |  |  |  |
| Age, years, median (IQR) | 63 (53-69.5) | 61 (54-70) | 63 (52-69) | .780 |
| Female sex | 52 (44.8) | 12 (34.3) | 40 (49.4) | .133 |
| BMI (n=169), kg/m², mean (SD) | 24.2 (5.9) | 24.9 (5.7) | 23.9 (5.9) | .387 |
| Surgical ward | 42 (36.2) | 18 (51.4) | 24 (29.6) | .025 |
| Medical ward | 74 (63.8) | 17 (48.6) | 57 (70.4) |  |
| Period |  |  |  |  |
| 2014-2017 | 41 (35.3) | 7 (20.0) | 34 (42.0) | .023 |
| 2018-2020 | 75 (64.7) | 28 (80.0) | 47 (58.0) |  |
| **Underlying conditions** |  |  |  |  |
| Solid tumor | 63 (54.3) | 21 (60.0) | 42 (51.9) | .419 |
| Hematologic malignancy | 6 (5.2) | 3 (8.6) | 3 (3.7) | .364 |
| Immunocompromised | 48 (41.4) | 11 (31.4) | 37 (45.7) | .153 |
| Palliative care | 6 (5.2) | 1 (2.9) | 5 (6.2) | .666 |
| CCWI, median (IQR) | 5 (3-8) | 6 (5-8) | 5 (2-7) | .273 |
| Permanent foreign device | 10 (8.6) | 4 (11.4) | 6 (7.4) | .486 |
| **Microbiological data** |  |  |  |  |
| Bacteremia duration (n=96), days, median (IQR) | 4 (2-6) | 4 (2-6) | 4 (2-6) | .641 |
| CoNS | 41 (35.3) | 14 (40.0) | 27 (33.3) | / |
| *S. aureus* | 31 (26.7) | 5 (14.3) | 26 (32.1) |  |
| GNB | 11 (9.5) | 3 (8.6) | 8 (9.9) |  |
| *Candida* spp. | 11 (9.5) | 5 (14.3) | 6 (7.4) |  |
| NFGNB | 4 (3.4) | 0 (0.0) | 4 (4.9) |  |
| Others | 18 (15.5) | 8 (22.9) | 10 (12.3) |  |
| **Biological data** |  |  |  |  |
| Creatinin, µmol/L, median (IQR) | 69 (51-94.5) | 67 (52-91) | 70 (50-97) | .674 |
| Maximal CRP (n=113), mg/L, median (IQR) | 113 (60-183) | 89  (60-178) | 117.5 (66.5-192.5) | .258 |
| **Clinical data** |  |  |  |  |
| Fever (n=104) | 101 (97.1) | 32 (100.0) | 69 (95.8) | / |
| Sepsis or septic shock | 24 (20.7) | 5 (14.3) | 19 (23.5) | .263 |
| ICU admission | 19 (16.4) | 6 (17.1) | 13 (16.0) | .884 |
| **Thrombosis** |  |  |  |  |
| Total thrombosis (n=95) | 9 (9.5) | 2 (6.7) | 7 (10.8) | .715 |
| >1 venous segment | 16 (13.8) | 4 (11.4) | 12 (14.8) | .774 |
| **Supplementary Table S2. *(continued)***  **Catheter** |  |  |  | ***(continued)*** |
| Type |  |  |  |  |
| Short-term CVC | 39 (33.6) | 19 (54.3) | 20 (24.7) | .008 |
| Long-term CVC | 36 (31.0) | 7 (20.0) | 29 (35.8) |  |
| Other catheters | 41 (35.4) | 9 (25.7) | 32 (39.5) |  |
| Duration (n=99), days, median (IQR) | 15 (8-37) | 19 (11-36) | 13 (7-37) | .155 |
| Indication |  |  |  |  |
| Parenteral nutrition | 24 (21.6) | 8 (23.5) | 16 (20.8) | .442 |
| Chemotherapy | 32 (28.8) | 7 (20.6) | 25 (32.5) |  |
| Other | 55 (49.6) | 19 (55.9) | 36 (46.7) |  |
| **Management** |  |  |  |  |
| Catheter removal | 114 (98.3) | 35 (100) | 79 (97.5) | / |
| AT duration, days, median (IQR) | 26 (21-33) | 19 (15-21) | 30 (25-42) | <.0001 |
| AT duration > 14 days | 109 (94.0) | 28 (80.0) | 81 (100.0) | / |
| Exclusive oral treatment | 1 (0.9) | 1 (2.9) | 0 (0.0) | / |
| Oral switch | 8 (6.9) | 1 (2.9) | 7 (8.6) | .432 |
| Curative anticoagulation (n=115) | 104 (90.4) | 29 (85.3) | 75 (92.6) | .297 |
| Anticoagulation duration (n=104) |  |  |  |  |
| ≤ 6 weeks | 30 (28.8) | 5 (17.2) | 25 (33.3) | .363 |
| > 6 weeks | 27 (26.0) | 8 (27.6) | 19 (25.3) |  |
| Long-term anticoagulation | 15 (14.4) | 6 (20.7) | 9 (12.0) |  |
| Unknown | 32 (30.8) | 10 (34.5) | 22 (29.4) |  |

Data are presented as numbers (%) unless otherwise indicated.

Abbreviations: AT: antimicrobial therapy; IQR: Interquartile range; BMI: body mass index; SD: standard deviation; CCWI: Charlson comorbidity weighted-index; CoNS: coagulase-negative staphylococci; GNB: Gram-negative bacilli; NFGNB: non-fermenting Gram-negative bacilli; CRP: C-reactive protein; ICU: intensive care unit; CVC: central venous catheter.

Supplementary Table S3. Risk factors of 30-day all-cause mortality or relapse in univariate analysis in patients with certain catheter-related septic deep venous thrombosis

|  | **Total**  **n=116** | **No death or relapse at D-30**  **n=100** | **Death or relapse at D-30**  **n=13** | **p value** |
| --- | --- | --- | --- | --- |
| **Treatment group** |  |  |  |  |
| Short-course | 35 (30.2) | 32 (32.0) | 3 (18.8) | .385 |
| Prolonged-course | 81 (69.8) | 68 (68.0) | 13 (81.3) |  |
| **Patient general status** |  |  |  |  |
| Age, years, median (IQR) | 63 (53-69.5) | 62.5 (53-69) | 67 (52-72.5) | .576 |
| Gender |  |  |  |  |
| Female | 52 (44.8) | 46 (46.0) | 6 (37.5) | .526 |
| Male | 64 (55.2) | 54 (54.0) | 10 (62.5) |  |
| BMI, kg/m², mean (SD) | 24.2 (5.9) | 24.4 (6) | 23.5 (5.1) | .553 |
| Hospital ward |  |  |  |  |
| Surgical ward | 42 (36.2) | 40 (40.0) | 2 (12.5) | **.034** |
| Medical ward | 74 (63.8) | 60 (60.0) | 14 (87.5) |  |
| Period |  |  |  |  |
| 2014-2017 | 41 (35.3) | 37 (37.0) | 4 (25.0) | .351 |
| 2018-2020 | 75 (64.7) | 63 (63.0) | 12 (75.0) |  |
| **Underlying conditions** |  |  |  |  |
| Solid tumor |  |  |  |  |
| Yes | 63 (54.3) | 52 (52.0) | 11 (68.8) | .212 |
| No | 53 (45.7) | 48 (48.0) | 5 (31.3) |  |
| Hematologic malignancy |  |  |  |  |
| Yes | 6 (5.2) | 6 (6.0) | 0 (0) | / |
| No | 110 (94.8) | 94 (94.0) | 16 (100) |  |
| Immunocompromised |  |  |  |  |
| Yes | 48 (41.4) | 40 (40.0) | 8 (50.0) | .451 |
| No | 68 (58.6) | 60 (60.0) | 8 (50.0) |  |
| Palliative care |  |  |  |  |
| Yes | 6 (5.2) | 2 (2.0) | 4 (25.0) | **.003** |
| No | 110 (94.8) | 98 (98.0) | 12 (75.0) |  |
| CCWI, median (IQR) | 5 (3-8) | 5 (2-8) | 7 (5.5-7.5) | .080 |
| Permanent foreign device |  |  |  |  |
| Yes | 10 (8.6) | 9 (9.0) | 1 (6.3) | 1 |
| No | 106 (91.4) | 91 (91.0) | 15 (93.8) |  |
| **Supplementary Table S3. *(continued)***  **Microbiological data** |  |  |  | ***(continued)*** |
| Bacteremia duration (n=96), days, median (IQR) | 4 (2-6) | 4 (2-6) | 4 (4-5) | .647 |
| Microorganism |  |  |  |  |
| *S. aureus* | 31 (26.7) | 28 (28.0) | 3 (18.8) | .663 |
| *Candida* spp. | 11 (9.5) | 10 (10.0) | 1 (6.2) |  |
| NFGNB | 4 (3.5) | 3 (3.0) | 1 (6.2) |  |
| Others | 70 (60.3) | 59 (59.0) | 11 (68.8) |  |
| **Biological data** |  |  |  |  |
| Creatinin, µmol/L, median (IQR) | 69 (51-94.5) | 66.5 (50-91.5) | 77.5 (59.5-112) | .337 |
| Maximal CRP (n=113), mg/L, median (IQR) | 113 (60-183) | 114 (60-184) | 92 (63-171) | .692 |
| **Clinical data** |  |  |  |  |
| Fever (n=104) |  |  |  |  |
| Yes | 101 (97.1) | 85 (96.6) | 16 (100) | / |
| No | 3 (2.9) | 3 (3.4) | 0 (0) |  |
| Sepsis or septic shock |  |  |  |  |
| Yes | 24 (20.7) | 19 (19.0) | 5 (31.2) | .318 |
| No | 92 (79.3) | 81 (81.0) | 11 (68.8) |  |
| ICU admission |  |  |  |  |
| Yes | 19 (16.4) | 16 (16.0) | 3 (18.8) | .725 |
| No | 97 (83.6) | 84 (84.0) | 13 (81.2) |  |
| **Thrombosis** |  |  |  |  |
| Total thrombosis (n=95) |  |  |  |  |
| Yes | 9 (9.5) | 9 (11.0) | 0 (0) | / |
| No | 86 (90.5) | 73 (89.0) | 13 (100) |  |
| >1 venous segment |  |  |  |  |
| Yes | 16 (13.8) | 12 (12.0) | 4 (25.0) | .232 |
| No | 100 (86.2) | 88 (88.0) | 12 (75.0) |  |
| **Catheter** |  |  |  |  |
| Type |  |  |  |  |
| Other catheters | 41 (35.4) | 34 (34.0) | 7 (43.8) | **.036** |
| Short-term CVC | 39 (33.6) | 38 (38.0) | 1 (6.2) |  |
| Long-term CVC | 36 (31.0) | 28 (28.0) | 8 (50.0) |  |
| Duration (n=99), days, median (IQR) | 15 (8-37) | 14 (8-32) | 29 (10-59) | .246 |
| Indication |  |  |  |  |
| Parenteral nutrition | 24 (21.6) | 21 (21.7) | 3 (21.4) | **.003** |
| Chemotherapy | 32 (28.8) | 23 (23.7) | 9 (64.3) |  |
| Other  **Supplementary Table S3. *(continued)*** | 55 (49.6) | 53 (54.6) | 2 (14.3) | ***(continued)*** |
| **Management** |  |  |  |  |
| Catheter removal |  |  |  |  |
| Yes | 114 (98.3) | 98 (98.0) | 16 (100) | / |
| No | 2 (1.7) | 2 (2.0) | 0 (0) |  |
| Time between the first microbiological documentation and catheter removal (n=167) | 2 (1-3) | 1 (1-3) | 3 (1-6.5) | **.037** |
| Time between the first microbiological documentation and initiation of effective AT | 1 (0-3) | 1 (0-3) | 1.5 (0-2.5) | .782 |
| AT duration, days, median (IQR) | 26 (21-33) | 26 (21-33) | 27 (23.5-35.5) | .752 |
| AT duration > 14 days |  |  |  |  |
| Yes | 109 (94.0) | 93 (93.0) | 16 (100) | / |
| No | 7 (6.0) | 7 (7.0) | 0 (0) |  |
| Exclusive oral treatment |  |  |  |  |
| Yes | 1 (0.9) | 1 (1.0) | 0 (0) | / |
| No | 115 (99.1) | 99 (99.0) | 16 (100) |  |
| Oral switch |  |  |  |  |
| Yes | 8 (6.9) | 6 (6.0) | 2 (12.5) | .304 |
| No | 108 (93.1) | 94 (94.0) | 14 (87.5) |  |
| Curative anticoagulation (n=115) |  |  |  |  |
| Yes | 104 (90.4) | 89 (89.9) | 15 (93.8) | 1 |
| No | 11 (9.6) | 10 (10.1) | 1 (6.3) |  |
| Anticoagulation duration (n=104) |  |  |  |  |
| ≤ 6 weeks | 30 (28.8) | 27 (30.3) | 3 (20.0) | .089 |
| > 6 weeks | 27 (26.0) | 26 (29.2) | 1 (6.7) |  |
| Long-term anticoagulation | 15 (14.4) | 12 (13.5) | 3 (20.0) |  |
| Unknown | 32 (30.8) | 24 (27.0) | 8 (53.3) |  |

Data are presented as numbers (%) unless otherwise indicated.

Abbreviations: IQR: Interquartile range; SD: standard deviation; BMI: body mass index; CCWI: Charlson comorbidity weighted-index; CRP: C-reactive protein; CoNS: coagulase-negative staphylococci; GNB: Gram-negative bacilli; NFGNB: non-fermenting Gram-negative bacilli; DVT: deep venous thrombosis; ICU: intensive care unit; CVC: central venous catheter; AT: antimicrobial therapy.

Supplementary Figure S1. Cumulative event curves for 90-day all-cause mortality in the unweighted (A) and inverse probability of treatment weighted (B) samples in total population.

|  |
| --- |

Supplementary Figure S2. Cumulative event curves for 90-day all-cause mortality in the unweighted (A) and inverse probability of treatment weighted (B) samples in patients with certain catheter-related bloodstream infection.



|  |
| --- |

Supplementary Figure S3. Cumulative event curves for 90-day relapse in the unweighted (A) and inverse probability of treatment weighted (B) samples in total population.

|  |
| --- |

Supplementary Figure S4. Cumulative event curves for 90-day relapse in the unweighted (A) and inverse probability of treatment weighted (B) samples in patients with certain catheter-related bloodstream infection.

|  |
| --- |
